# Exploring cognitive impairments and the efficacy of phosphatidylcholine and computer-assisted cognitive training in post-acute COVID-19 and post-acute COVID-19 Vaccination Syndrome

**DOI:** 10.3389/fneur.2024.1419134

**Published:** 2024-09-03

**Authors:** Julian Frederic Hotz, Sophie Kellerberger, Sara Elea Jöchlinger, Iren Danielova, Hanife Temizsoy, Sandra Ötsch, Jürgen Goller, Muhammad Yacob, Udo Zifko

**Affiliations:** ^1^Department of Neurology, Evangelic Hospital Vienna, Vienna, Austria; ^2^Department of Neurology, Hospital St. John's of God, Vienna, Austria; ^3^Department of Medicine I, Division of Infectious Diseases and Tropical Medicine, Medical University of Vienna, Vienna, Austria; ^4^Karl Landsteiner University of Health Sciences, Krems, Austria; ^5^Department of Neurology, Rudolfinerhaus Private Clinic and Campus, Vienna, Austria; ^6^Department of Clinical Psychology, Rudolfinerhaus Private Clinic and Campus, Vienna, Austria; ^7^Department of Neurology, Accident Hospital Meidling, Vienna, Austria

**Keywords:** SARS-CoV-2, COVID-19, Post-acute COVID-19 Syndrome, Post-acute COVID-19 Vaccination Syndrome, phosphatidylcholine, computer-assisted cognitive training, WHOQOL-BREF

## Abstract

**Purpose:**

The COVID-19 pandemic has led to millions of confirmed cases worldwide, resulting in numerous deaths and hospitalizations. Long-term symptoms after infection or vaccination, known as Post-acute COVID-19 Syndrome (PACS) or Post-acute COVID-19 Vaccination Syndrome (PACVS), present a challenge for the healthcare system. Among the various neurological symptoms, cognitive impairments are frequently observed in PACS/PACVS patients. This study aimed to understand cognitive deficits in PACS/PACVS patients and evaluated potential treatment options, including phosphatidylcholine and computer-assisted cognitive training (CCT).

**Methods:**

The Neuro-COVID Outpatient Clinic at Evangelic Hospital Vienna evaluated *n* = 29 PACS/PACVS patients from May 2023 to October 2023. Enrolled patients were divided into three therapy schemes: Group A received phosphatidylcholine, B received phosphatidylcholine plus access to a computer-assisted cognitive training program, and C (divided into two subgroups) served as a control group. Cognitive impairments were evaluated in multiple assessments (initial and during therapy) using the COGBAT test. Simultaneously, an assessment of the quality of life was conducted using the WHOQOL-BREF.

**Results:**

Primary cognitive impairments, especially attentional deficits were notably evident compared to the general population. While all treatment groups showed cognitive improvement (significant or with a positive trend, but without reaching the level of statistical significance) after therapy, no significant interaction was found between assessment time points and treatment schemes for overall cognitive performance, attention, memory, and executive functions, suggesting consistency across the groups. The WHOQOL-BREF primarily demonstrated deficits in the domains of physical health and psychological well-being.

**Conclusion:**

This study examined the impact of PACS/PACVS on cognitive performance and evaluated phosphatidylcholine and CCT as potential treatment options. Patients with PACS/PACVS showed notable cognitive deficits, especially in the domain attention. While the effectiveness of phosphatidylcholine and CCT in treating cognitive deficits was inconclusive, the study indicated the possibility of spontaneous remission of cognitive deficits in PACS/PACVS.

## 1 Introduction

According to the World Health Organization (WHO), there have been more than 750 m. confirmed cases of an infection with SARS-CoV-2 worldwide until 03/2024 (1). The majority of individuals who tested positive achieved full recovery. Nevertheless, at least 7 m. individuals died due to COVID-19, and a substantial number of patients required hospitalization including intensive care. Beyond the acute COVID-19 disease, the emergence of long-term symptoms poses an additional significant problem. The WHO and the National Institute for Health and Care Excellence (NICE) have established clinical case definitions, which were later terminologically denoted as Post-acute COVID-19 Syndrome (PACS) (2–4). PACS is defined as symptoms persisting for at least 12 weeks after an acute SARS-CoV-2 infection. Furthermore, Post-acute COVID-19 Vaccination Syndrome (PACVS) has also been described. While it has been demonstrated that SARS-CoV-2 vaccinations may have prevented around 14.4 million deaths across 185 countries in the initial vaccination year, there has been an emergence of vaccine-associated adverse effects in the following months and years (5–9).

Frequently observed neurological symptoms in PACS include persistent fatigue, cognitive impairments, headache, postural orthostatic tachycardia syndrome (POTS), mood fluctuations, sleeping disorders, muscular weakness, myalgia and persistent loss of taste or smell (4, 10–17). This study primarily focuses on the cognitive impairments of patients following SARS-CoV-2 infection or vaccination. Neurocognition can be categorized into the six main domains attention, executive functions, learning and memory, language, perceptual-motor functions, and social cognition (18). In people with previous SARS-CoV-2 infections the main affected domains were attention, executive functions, learning, and memory (19–26). The language domain was less affected, but nevertheless deficits were observed, including in receptive and expressive language (27, 28). It should further be noted that previous studies on PACS patients have demonstrated discrepancies between subjectively perceived cognitive impairments and results from standardized neuropsychological testing procedures (23, 29).

The treatment of PACS or PACVS remains a challenge for physicians in hospital and outpatient clinics. Numerous patients exhibit a wide spectrum of symptomatology, necessitating individualized treatment approaches (10, 11). Personalized rehabilitation programs might be a promising opportunity for the treatment of PACS (30, 31). In literature, a score of 2 or higher on the Post-COVID-19 Functional Status Scale indicates the need for neurological rehabilitative measures (32). Prior studies have demonstrated that rehabilitation programs can ameliorate many symptoms, whereas the treatment of respiratory and neurological symptoms is often challenging (33, 34). Several medications are being investigated in larger trials for the pharmacological treatment of neurological symptoms in PACS. To date, there are no confirmatory trials on treatment strategies. Regarding the pharmacological treatment of cognitive deficits, only limited data are available (35). For this reason, novel therapeutic approaches involving phosphatidylcholine and computer-assisted cognitive training (CCT) were explored as treatment options for cognitive impairments associated with PACS or PACVS. Phosphatidylcholine is a mixture of neutral lipids and phospholipids, which are essential components of the central nervous system, especially of the cellular membranes. This natural emulsifier is synthesized by both plants (e.g., soya) and animals (e.g., egg yolk). Phosphatidylcholine is a precursor of choline, which is metabolized into acetylcholine, one of the most important neurotransmitters involved in memory and learning (36). Phosphatidylcholine might enhance neuronal development (37). It has demonstrated positive effects on learning and memory performance in healthy participants and in patients with dementia or cerebrovascular diseases (38–43). Further, phosphatidylcholine might decrease the risk of developing APOE4-associated Alzheimer’s disease (44). Due to insufficient inpatient and outpatient rehabilitation infrastructure for cognitive deficits associated with PACS or PACVS, hope is also being placed on the pharmacological ‘pill plus’ approach. This approach combines pharmacological treatment with computer-assisted neurorehabilitation of cognitive deficits. Since the early 1980s, CCT has been available as an adjunct for speech therapists in inpatient facilities and also, although less commonly, as a tool for patient-directed therapy at home (45). CCT aims to improve deficient brain functions through repetitive practice, thereby enhancing neural networks. Studies have demonstrated the efficacy of CCT for cognitive impairments in neurorehabilitation, especially after strokes (46–49). This prospective study aimed to evaluate cognitive deficits in patients with PACS and PACVS and to improve therapeutic options for these conditions. Since PACS or PACVS patients may show comparable cognitive impairments to degenerative or vascular dementia, as described above, this study examined whether the positive effects of phosphatidylcholine and CCT in dementia can also be transferred to PACS and PACV.

## 2 Materials and methods

### 2.1 Method/procedure

**Figure 1:**
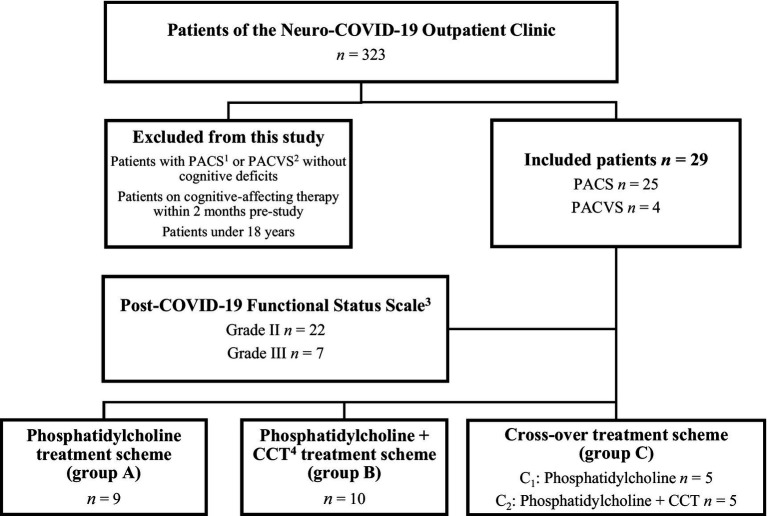
Grouping of patient collectives according to treatment schemes. ^1^Post-acute COVID-19 Syndrome, ^2^Post-acute COVID-19 Vaccination Syndrome, ^3^Klok et al. (32), ^4^Computer-assisted cognitive training.

In response to the increasing number of PACS or PACVS patients with new suspected neurological symptoms worldwide, the Department of Neurology at the Evangelic Hospital Vienna established a Neuro-COVID Outpatient Clinic in June 2021 to provide comprehensive systematic assessment and care for these patients. Until November 2023, a total of 323 patients (06–12/2021: 57, 2022: 140, 01–11/2023: 126) presenting with suspected PACS or PACVS underwent examination. From this cohort, *n* = 29 patients with subjectively perceived cognitive deficits and first appointment between May 01, 2023 and October 31, 2023 were included in this prospective study. Exclusion criteria were PACS or PACVS patients without cognitive deficits, patients who received cognitive-affecting therapy within 2 months pre-study and patients under 18 years of age. Selection and comprehensive examination of the included patients was performed at the first visit by an independent specialist in neurology in order to confirm the PACS or PACVS diagnosis using the NICE criteria (2). All included patients provided informed written consent. After approval, the included patients completed two questionnaires about their demographic data and another about their quality of life. According to the given answers in the questionnaires about PACS or PACVS symptoms, the grade of the disease was classified by the Post-COVID-19 Functional Status Scale (32). To evaluate the quality of life, the Quality of Life (QOL) assessment WHOQOL-BREF was used (50, 51). Subsequently, neuropsychological testing was conducted using the COGBAT test set (Schuhfried GmbH, Mödling, Austria). Following this, patients were randomly categorized into one of three groups. Patients were allocated using a randomized computer-based algorithm and a concealed allocation generated by the first author using https://www.randomizer.org. The results of the randomization were kept in non-transparent covers. The randomizer, who performed and communicated the group allocation, was not involved in the neuropsychological testing. The patients were aware of which group they were categorized to. Neuropsychological testing was performed by a psychologist who did not know which group each patient belonged to. Since PACS or PACVS symptoms can vary during the course of the day, the best possible care was taken to arrange the follow-up checks at the same times as initial presentation. Other potential confounders such as excessive noise, heat, air quality or lighting were kept to a minimum by using the same room for all patients included in the study. Patients in group A received 22.5 mL liquid phosphatidylcholine (Buerlecithin®, Orifarm GmbH, Healthcare A/S, Odense S, Denmark) three times daily for 4 weeks after the initial neuropsychological testing, followed by a re-evaluation using COGBAT test set and WHOQOL-BREF. Patients in group B, in addition to the 4-week treatment scheme of phosphatidylcholine (22.5 mL three times daily), received access to the myReha app (nyra health GmbH, Vienna, Austria) for 4 weeks with a minimum daily training duration of 25 min (Monday to Friday). After this combined 4-week “pill plus” approach of phosphatidylcholine + CCT, a follow-up using COGBAT test set and WHOQOL-BREF was performed. Group C (cross-over treatment scheme) received no therapy for 4 weeks after the initial neuropsychological testing, followed by re-evaluation using COGBAT test set and WHOQOL-BREF. Subsequently, the patients received the treatments and re-evaluations of group A (group C_1_) or B (group C_2_) (Figure 1).

### 2.2 Cognitive testing

The test set COGBAT is used for the evaluation of cognitive performance, encompassing the assessment of three neurocognitive domains including attention, memory, and executive functions. Each of these domains is divided into three subdimensions. The attention assessment included tests for alertness, divided attention, and processing speed. Memory performance was assessed through tests for learning ability, short- and long-term recall, and recognition of geometric figures. Executive functions were evaluated based on cognitive flexibility, planning ability, and working memory. The test battery consists of two parallel versions (S1 and S2), making it well-suited for follow-up assessments. The testing is computer-based and typically takes about 1 h to complete. This comprehensive and time-efficient evaluation of cognitive status is entirely independent of test administration (52).

### 2.3 Computer-assisted cognitive training (CCT)

For CCT, the app MyReha was used, which is an authorized app for use in cognitive impairments with class I medical device CE certification. The app is a digital, German-language tablet-based neurorehabilitation platform for patients with neurocognitive deficits, providing comprehensive evidence-based therapy across a range of cognitive domains. It is based on the principle of “retraining,” based on the assumption that cognitive performance can be improved through repeated stimulation, such as frequently practiced exercises (53). This repetition is believed to support brain function restoration through changes in synaptic connectivity (54, 55). Through the myReha app, patients receive a personalized weekly plan tailored to their current strengths and weaknesses in attention, memory, language, and executive function. By employing artificial intelligence solutions, the app achieves a high degree of individualization, which is a critical aspect of neurorehabilitation, and is accessible anytime and anywhere. The myReha app’s therapy content adheres to the therapeutic gold standard, aligning with current guidelines of the German Society for Neurorehabilitation, and is developed by a therapy team with extensive clinical experience in patients with cognitive deficits (56).

### 2.4 WHOQOL-BREF

To assess quality of life, the WHOQOL-BREF, a modified version of the WHOQOL-100 comprising 26 questions, was utilized (50, 51). Responses collected were aggregated into sum scores for the domains of physical health (7 items), psychological well-being (6 items), social relationships (3 items), and environmental health (8 items). Patients provided scores ranging from 1 to 5 for each response option. Raw scores within each domain were transformed to a scale range of 0–100, with 100 representing the highest possible quality of life.

### 2.5 Ethics statement

The primary objective of this study was to evaluate and potentially enhance the cognitive performance of patients with PACS and PACVS. The Ethics Committee of the Evangelic Hospital Vienna approved the observational study under protocol number 03/2023 on April 25, 2023.

### 2.6 Statistics

Data analysis was conducted in a pseudonymous manner using IBM SPSS Statistics Version 29.0 (IBM). For comparative statistics, a 95.0% confidence interval and a significance level (*α*) of 0.05 were established. The alternative hypothesis was accepted when the calculated *p*-value was less than *α*. Categorical data was presented with absolute frequencies and their percentage representation within the total sample. Metric variables were summarized using mean, minimum, and/or maximum values. Normality of metric variables was assessed through the Kolmogorov–Smirnov and Shapiro–Wilk tests. Group comparisons employed parametric tests when normal distribution was confirmed, and non-parametric tests when normal distribution was not established. Parametric tests included dependent or independent sample t-tests, while non-parametric tests involved Wilcoxon and sign tests for dependent variables, and the Mann–Whitney U test for independent variables. To investigate differences in cognitive functions according to measurement time points and treatment schemes, repeated measures ANOVA was used. For correlations, either Pearson or Spearman’s rank correlation coefficient (*K*) was used depending on the normal distribution or scaling of the variables. Cronbach’s alpha was employed to assess the internal consistency of scales, with values between 0.60 and less than 0.80 considered adequate, those between 0.80 and less than 0.85 regarded as good, and values of 0.85 or higher deemed excellent.

## 3 Results

### 3.1 Study population and disease-specific data

The 29 enrolled patients were divided into 19 (65.5%) women and 10 (34.5%) men (f/m = 1.9). The mean age was *M* = 44.8 ± 12.8 years (21.7–66.5 years). For 13 patients (44.8%), the highest level of education was a university degree, for 10 (34.5%) a high school diploma, for 3 (10.3%) a middle school diploma, for 2 (6.9%) an apprenticeship diploma, and for 1 patient (3.4%) a compulsory school diploma.

In total, 25 patients diagnosed with PACS and 4 with PACVS were enrolled in the study. Out of the 25 PACS patients, the causative virus variant remained unknown in 11 patients (44.0%). SARS-CoV-2 Omicron (B1.1.529) variant was identified in 9 (36.0%), Delta (B.1.617.2) variant in 3 (12.0%), and Beta (B.1.351) variant in 2 (8.0%) patients. 17 patients (68.0%) had received three doses of vaccination at the time of infection, 3 (12.0%) had received two doses, and 1 (4.0%) had received four doses. 4 (16.0%) patients were not vaccinated. Out of these 4 patients, two were the only patients with severe acute infections. The majority of patients (21/25, 84.0%) experienced mild symptoms during the acute SARS-CoV-2 infection. None of the included individuals reported a critical clinical course (acute respiratory distress syndrome, multiorgan failure, and/or mechanical ventilation). According to the Post-COVID-19 Functional Status Scale, 22 patients (75.9%) were classified as grade II and 7 patients (24.1%) as grade III, indicating the recommendation for rehabilitative measures for all included patients (32). 13 (44.8%) patients had neurological or psychiatric comorbidities prior to PACS or PACVS. The described comorbidities included depression in 7 (24.1%), migraine in 6 (20.7%), and tension headaches in 5 (17.2%) patients, and bipolar disorder (3.5%), pre-existing meningioma (3.5%), and post-traumatic stress disorder in one patient each (3.5%) (Table 1).

**Table 1:** Demographic data of *n* = 29 patients with PACS or PACVS.

Demographic data	Value^1^
Cohort characteristics	
Female	19 (65.5%)
Male	10 (34.5%)
Age	44.8 ± 12.8 (21.7–66.5)
Education	
University degree	13 (44.8%)
High school diploma	10 (34.5%)
Middle school diploma	3 (10.3%)
Apprenticeship diploma	2 (6.9%)
Compulsory school diploma	1 (3.4%)
Neurological or psychiatric comorbidities	
Depression	7 (24.1%)
Migraine	6 (20.7%)
Tension headaches	5 (17.2%)
Bipolar disorder	1 (3.5%)
Meningioma	1 (3.5%)
Post-traumatic stress disorder	1 (3.5%)
Disease specific data	
Post-acute COVID-19 Syndrome (PACS)	25 (86.2%)
SARS-CoV-2 Omicron (B1.1.529)	9 (36.0%)
SARS-CoV-2 Delta (B.1.617.2)	3 (12.0%)
SARS-CoV-2 Beta (B.1.351)	2 (8.0%)
Unknown	11(44.0%)
Four doses of vaccination^2^	1 (4.0%)
Three doses of vaccination^2^	17 (68.0%)
Two doses of vaccination^2^	3 (12.0%)
Unvaccinated^2^	4 (16.0%)
Post-acute COVID-19 Vaccination Syndrome (PACVS)	4 (13.8%)
SARS-CoV-2/COVID-19 acute disease course	
Severe acute infection	4 (16.0%)
Mild symptoms	21 (84.0%)
Post-COVID-19 Functional Status Scale^3^	
Grade II	22 (75.9%)
Grade III	7 (24.1%)

^1^Absolute number (%) or Mean ± standard deviation (interquartile range), ^2^Vaccination status at time of infection, ^3^Classificated according to Klok et al. (32).

**Figure 2:**
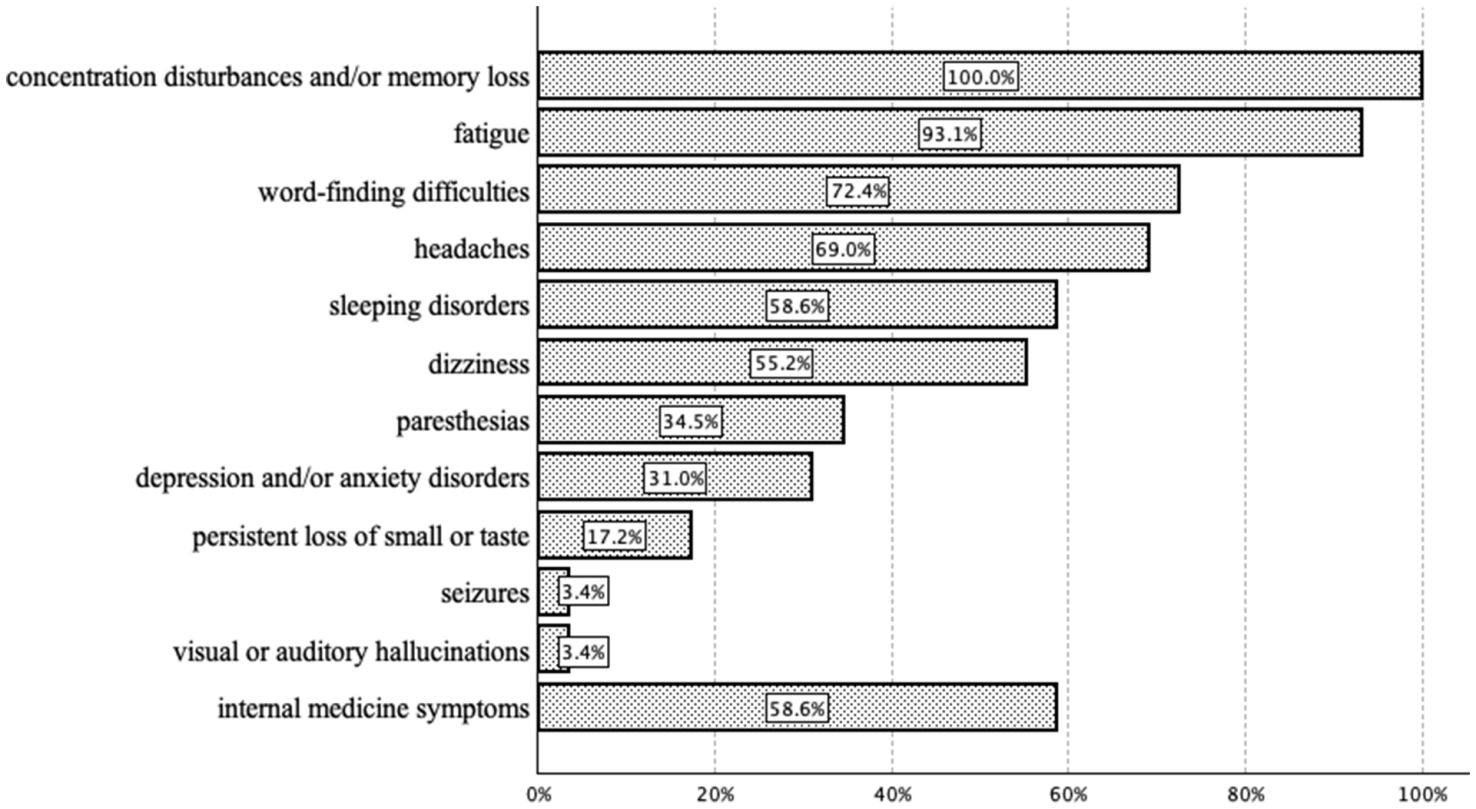
Reported new onset symptoms at least 12 weeks after infection with SARS-CoV-2 in *n* = 29 PACS or PACVS patients.

At the time of initial presentation, the included patients reported experiencing symptoms of PACS or PACVS for an average of *M* = 12.8 ± 7.2 months. Consistent with the inclusion criteria, all patients reported concentration disturbances and/or memory loss. Additionally, as new onset neurological or psychiatric symptoms of PACS or PACVS, 27 (93.1%) patients exhibited fatigue, 21 (72.4%) word-finding difficulties, 20 (69.0%) headaches, 17 (58.6%) sleep disorders, 16 (55.2%) dizziness, 10 (34.5%) paresthesias, 9 (31.0%) depression and/or anxiety disorders, 5 (17.2%) persistent loss of smell or taste, 1 (3.4%) visual or auditory hallucinations, and 1 (3.4%) a seizure (Figure 2). Furthermore, 17 (58.6%) participants reported experiencing internal medical sequelae such as palpitations (10, 34.5%) and/or respiratory distress (9, 31.0%). 21 (72.4%) patients reported being less physically active due to their symptoms.

### 3.2 Cognitive testing

When examining the descriptive statistics of the COGBAT test results at the initial measurement time point, it became evident that below average results (*t* < 50) are observed across nearly all dimensions compared to the general population (Table 2).

**Table 2:** COGBAT test results of *n* = 29 patients with PACS or PACVS.

COGBAT	Min	Max	Average	SD^1^
Cognitive performance	20	69	45.07	10.10
Attention	20	56	39.55	9.78
Subdimension: alertness, visual	20	60	39.17	8.46
Subdimension: divided attention	0	63	44.39	12.51
Subdimension: processing speed	27	60	43.00	8.47
Memory	31	71	49.62	10.15
Subdimension: learning ability	27	68	50.38	11.09
Subdimension: short-term recall	31	60	48.55	9.80
Subdimension: long-term recall	37	61	49.68	8.16
Subdimension: recognition	35	60	49.11	8.59
Executive functions	29	69	47.48	8.77
Subdimension: cognitive flexibility	32	65	48.38	7.80
Subdimension: planning ability	31	65	50.64	8.82
Subdimension: working memory, verbal	27	61	45.14	9.63

^1Standard deviation.^

**Figure 3:**
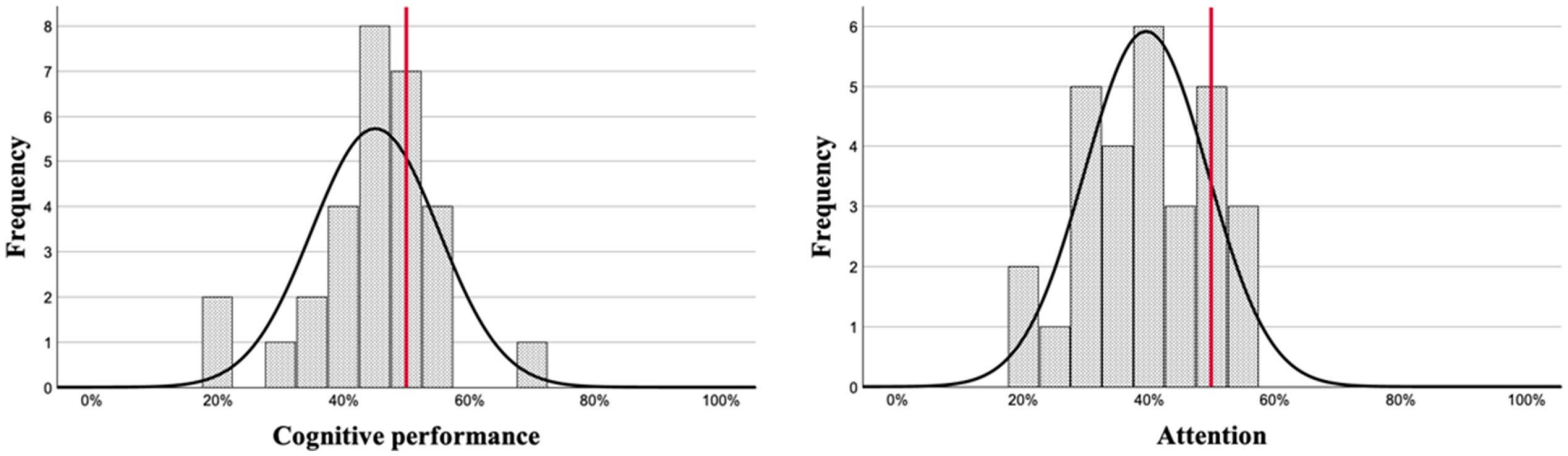
COGBAT test results of cognitive performance (left) and attention (right) of *n* = 29 PACS or PACVS patients compared to the general population (red line).

Foremost, a deficiency in the domain of attention (*M* = 39.55 ± 9.78) was evident when compared to the general population. There was also a decline observed in overall cognitive performance (*M =* 45.07 ± 10.10) compared to the general population (Figure 3).

Following the last neuropsychological assessment, after completion of the 4- or 8-week treatment phase, 14 patients (48.3%) reported subjectively perceiving cognitive improvement. Based on treatment scheme, 33.3% of group A and 40.0% of group B subjectively reported cognitive improvement after 4 weeks. Within the control groups, 60.0% of the patients in the phosphatidylcholine scheme (C_1_) and 80.0% of the phosphatidylcholine + CCT (C_2_) scheme reported subjective improvement in cognitive performance.

In COGBAT testing, group A and B showed significant (*p*_A_ = 0.011, *p*_B_ = 0.031) improvement in cognitive performance from first to second time point. Significant improvement in cognitive performance was also observed in group C_1_ from first to second (*p*_C1_ = 0.009), second to third (*p*_C1_ = 0.037) and first to third assessment (*p*_C1_ = <0.001). A positive trend, which however did not reach the level of statistical significance, in cognitive performance was observed in group C_2_ from first to second (*p*_C2_ = 0.053), second to third (*p*_C2_ = 0.090) and first to third (*p*_C2_ = 0.058) assessment. Furthermore, improvements were also noted in attention, memory, and executive functions in all treatment schemes (Table 3).

**Table 3:** Results of COGBAT testing in *n* = 29 PACS or PACVS patients based on treatment schemes.

Dimension	Treatment scheme	Assessment	Mean ± SD^1^	*p-*value
Cognitive performance	A (*n* = 9)	1	48.4 ± 12.953	0.011
		2	53.4 ± 15.339	
	B (*n* = 10)	1	44.80 ± 6.779	0.031
		2	50.70 ± 8.301	
	C_1_ (*n* = 5)	1	45.40 ± 6.656	0.009 0.037
		2	50.40 ± 5.595	
		3	54.00 ± 5.385	
	C_2_ (*n* = 5)	1	39.20 ± 12.716	0.053 0.090
		2	47.80 ± 6.907	
		3	50.40 ± 5.814	
Attention	A (*n* = 9)	1	42.44 ± 13.049	0.029
		2	47.33 ± 12.884	
	B (*n* = 10)	1	38.90 ± 7.047	0.108
		2	43.70 ± 7.119	
	C_1_ (*n* = 5)	1	43.80 ± 5.263	0.926 0.855
		2	44.00 ± 4.528	
		3	44.60 ± 2.793	
	C_2_ (*n* = 5)	1	31.40 ± 7.987	0.100 0.229
		2	38.80 ± 6.261	
		3	41.40 ± 3.286	
Memory	A (*n* = 9)	1	53.67 ± 10.173	0.179
		2	57.78 ± 12.163	
	B (*n* = 10)	1	47.50 ± 9.046	0.038
		2	53.20 ± 9.762	
	C_1_ (*n* = 5)	1	47.40 ± 15.339	0.006 0.205
		2	57.80 ± 14.704	
		3	64.60 ± 14.276	
	C_2_ (*n* = 5)	1	48.80 ± 6.301	0.116 0.491
		2	54.80 ± 7.887	
		3	57.80 ± 13.517	
Executive functions	A (*n* = 9)	1	49.67 ± 10.618	0.013
		2	56.22 ± 9.284	
	B (*n* = 10)	1	47.80 ± 6.596	0.015
		2	54.70 ± 8.070	
	C_1_ (*n* = 5)	1	47.80 ± 5.891	0.113 0.175
		2	51.40 ± 6.465	
		3	54.80 ± 8.526	
	C_2_ (*n* = 5)	1	42.60 ± 11.803	0.100 0.002
		2	47.80 ± 11.032	
		3	52.40 ± 11.675	

^1standard deviation.^

A negative correlation (*K* = −0.489, *p* = 0.007) was observed between the number of months with PACS or PACVS and the results of cognitive performance in the COGBAT testing. The duration of PACS or PACVS in months did not significantly correlate (*K* = 0.31, *p* = 0.099) with the improvement of cognitive deficits from initial to second assessment.

The ANOVA revealed no significant interaction between the assessment time point and treatment scheme concerning cognitive performance (*F* (3, 25) = 0.46, *p* = 0.714). Similarly, no significant interaction was found for attention (*F* (3, 25) = 0.94, *p* = 0.438), memory (*F* (3, 25) = 0.82, *p* = 0.493), and executive functions (*F* (3, 25) = 0.37, *p* = 0.775). Examining the main effects concerning cognitive performance, a significant within-subject effect was observed (*F* (1, 25) = 28.22, *p* < 0.001, *η_p_2* = 0.53). Similarly, within attention (*F* (1, 25) = 10.05, *p* = 0.004, *η_p_2* = 0.29), memory (*F* (1, 25) = 21.42, *p* < 0.001, *η_p_2* = 0.46), and executive functions (*F* (1, 25) = 21.08, *p* < 0.001, *η_p_2* = 0.46) significant within-subject effects were noted. Thus, differences in the cognitive domains of cognitive performance, attention, memory, and executive functions were observed, which can be attributed to the assessment time point, regardless of which treatment scheme the patients received between the first and second assessment time points. An additional repeated measures ANOVA was used for the two control groups (C_1_ and C_2_). Hereby, the ANOVA revealed no significant interaction between the assessment time point and treatment scheme concerning cognitive performance (*F* (1.31, 10.47) = 0.75, *p* = 0.443), attention (*F* (1.85, 14.81) = 2.82, *p* = 0.095), memory (*F* (1.51, 12.09) = 1.43, *p* = 0.270), and executive functions (*F* (1.75, 14) = 0.53, *p* = 0.574). Upon examination of the main effects, significant within-subject effects concerning cognitive performance (F (1.31, 10.47) = 22.15, *p* < 0.001, *η_p_2* = 0.74), memory (*F* (1.51, 12.09) = 14.88, *p* < 0.001, *η_p_2* = 0.65), and executive functions (*F* (1.75, 14) = 19.12, *p* < 0.001, *η_p_2* = 0.71) became apparent. In the domain attention, no significant within-subject effect was observed (*F* (1.85, 14.81) = 3.70, *p* = 0.052). Thus, differences were noted in the domains cognitive performance, memory, and executive functions, which can be linked to the assessment time point, regardless of the treatment scheme between the second and third assessment time points.

A correlation emerged between the duration of PACS or PACVS in months and the onset of depressive and/or anxiety symptoms (*K* = 0.44, *p* = 0.017). The COGBAT test results concerning cognitive performance (*K* = −0.37, *p* = 0.048) and executive functions (K = −0.38, *p* = 0.040) displayed negative correlations with the symptoms of depression and anxiety. No correlation was found (*K* = 0.02, *p* = 0.908) between the symptoms of depression and/or anxiety and the improvement in cognitive performance on the COGBAT test. A negative correlation (*K* = −0.63, *p* < 0.001) was observed between age and cognitive performance. Additionally, a correlation (*K* = 0.41, *p* = 0.025) was found between age and the reporting of depressive and/or anxiety symptoms. Furthermore, a negative correlation became apparent between the presence of fatigue and cognitive performance (*K* = −0.40, *p* = 0.032), particularly in the domain of attention (*K* = −0.39, *p* = 0.036). No significant correlation (*K* = 0.18, *p* = 0.353) was found between age and fatigue.

### 3.3 Results of the WHOQOL-BREF

**Figure 4:**
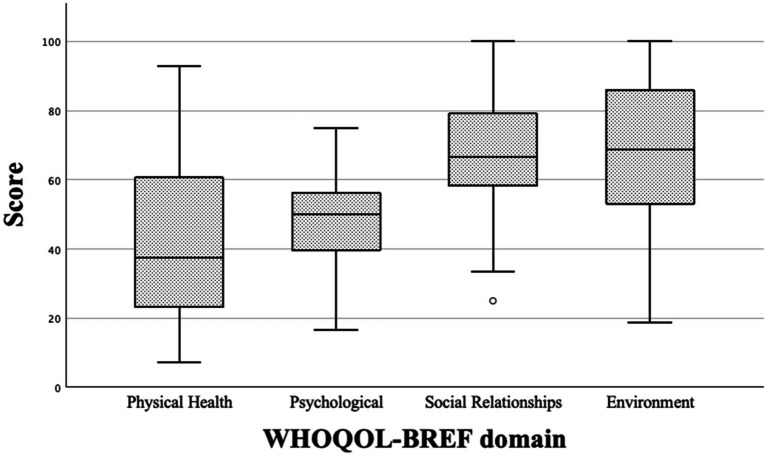
Boxplots of the WHOQOL-BREF domain scores from *n* = 29 patients with PACS or PACVS.

Using the WHOQOL-BREF, 25 out of 29 patients (86.2%) reported being very dissatisfied or dissatisfied with their health status. In the domains of physical health and psychological health, average WHOQOL-BREF scores were *M =* 41.3 ± 22.2 and 47.5 ± 14.1, respectively. Average scores for the social relationships and environment domains were *M =* 64.9 ± 19.0 and 68.4 ± 20.2, respectively (Figure 4). The WHO quality of life exhibited good reliability across all scored items (Cronbach’s alpha coefficient 0.93). This reliability persisted upon analyzing the QOL domains separately, revealing coefficients of 0.66 for physical health, 0.79 for psychological, 0.60 for social relationships, and 0.92 for the environmental. The domains of psychological health and social relationships showed a negative correlation (*K*_1_ = −0.43, *p* = 0.021; *K*_2_ = −0.552, *p* = 0.002) with the presence of depression in the patient’s medical history.

Overall, 18 out of 28 patients (64.2%) reported insufficient energy for daily life activities. Half of these patients (14 out of 18) indicated experiencing little to no enjoyment in life. Additionally, 23 out of 28 patients (82.14%) reported frequently experiencing negative emotions such as feeling blue, despair, anxiety, or depression (Table 4).

**Table 4:** WHOQOL-BREF results of *n* = 29 patients with PACS or PACVS.

Scale points domains and facets	*n*	1 Poor QOL^1^	2	3	4	5 Good QOL^1^
General QOL	29	2 (6.9%)	7 (24.1%)	15 (51.7%)	5 (17.2%)	0 (0%)
General health	29	6 (20.7%)	19 (65.5%)	3 (10.3%)	1 (3.4%)	0 (0%)
Physical health						
Pain and discomfort	28	5 (17.9%)	7 (25.0%)	6 (21.4%)	8 (28.6%)	2 (7.1%)
Energy and fatigue	28	9 (32.1%)	9 (32.1%)	6 (21.4%)	4 (14.3%)	0 (0%)
Sleep and rest	28	4 (14.3%)	12 (42.9%)	6 (21.4%)	3 (10.7%)	3 (10.7%)
Dependence on medication	28	6 (21.4%)	6 (21.4%)	7 (25.0%)	7 (25.0%)	2 (7.1%)
Mobility	28	1 (3.6%)	6 (21.4%)	8 (28.6%)	8 (28.6%)	5 (17.9%)
Activities of daily living	28	9 (32.1%)	9 (32.1%)	3 (10.7%)	5 (17.9%)	2 (7.1%)
Work capacity	28	17 (60.7%)	6 (21.4%)	3 (10.7%)	2 (7.1%)	0 (0%)
Psychological health						
Positive feelings	28	4 (14.3%)	10 (35.7%)	12 (42.9%)	2 (7.1%)	0 (0%)
Negative feelings	28	0 (0%)	5 (17.9%)	12 (42.9%)	11 (39.3%)	0 (0%)
Self-esteem	28	1 (3.6%)	6 (21.4%)	6 (21.4%)	10 (36.7%)	5 (17.9%)
Thinking, learning, memory and concentration	28	4 (14.3%)	15 (53.6%)	8 (28.6%)	1 (3.6%)	0 (0%)
Body image	28	0 (0%)	3 (10.7%)	6 (21.4%)	15 (53.6%)	4 (14.3%)
Satisfy with you	26	1 (3.8%)	6 (23.1%)	11 (42.3%)	7 (26.9%)	1 (3.8%)
Social Relationships						
Personal relations	27	0 (0%)	4 (14.8%)	3 (11.1%)	13 (48.1%)	7 (25.9%)
Sex	28	3 (10.7%)	4 (14.3%)	9 (32.1%)	8 (28.6%)	4 (14.3%)
Practical social support	27	0 (0%)	2 (7.4%)	4 (14.8%)	13 (48.1%)	8 (29.6%)
Environment						
Financial resources	28	2 (7.1%)	3 (10.7%)	4 (14.3%)	9 (32.1%)	10 (35.7%)
Information and skills	27	0 (0%)	0 (0%)	6 (22.2%)	9 (33.3%)	12 (44.4%)
Recreation and leisure	28	2 (7.1%)	7 (25.0%)	9 (32.1%)	5 (17.9%)	5 (17.9%)
Home environment	28	1 (3.6%)	1 (3.6%)	2 (7.1%)	13 (46.4%)	11 (39.3%)
Access to health and social care	28	2 (7.1%)	6 (21.4%)	3 (10.7%)	13 (46.4%)	4 (14.3%)
Physical safety and security	28	2 (7.1%)	3 (10.7%)	12 (42.9%)	9 (32.1%)	2 (7.1%)
Physical environment	28	0 (0%)	1 (3.6%)	5 (17.9%)	8 (28.6%)	14 (50.0%)
Transport	27	0 (0%)	4 (14.8%)	4 (14.8%)	6 (22.2%)	13 (48.1%)

^1Quality of life.^

Significant improvement in the physical health domain was observed in group A from the first to the second assessment point (*p*_A_ = 0.032). However, in all other investigated groups, there was no significant progress in the physical health domain from the first to the second assessment point (*p*_B_ = 0.330, *p*_C1_ = 0.370, *p*_C2_ = 0.682) or the third assessment point (*p*_C1_ = 0.097, *p*_C2_ = 0.389). There was no significant improvement in the psychological domain in any of the investigated groups from the first to the second assessment point (*p*_A_ = 0.169, *p*_B_ = 0.402, *p*_C1_ = 0.174, *p*_C2_ = 0.710) or the third assessment point (*p*_C1_ = 0.066, *p*_C2_ = 0.367). Similarly, there was no significant progress in any of the investigated groups in the domain social relationships from the first to the second assessment point (*p*_A_ = 0.512, *p*_B_ = 0.680, *p*_C1_ = 0.529, *p*_C2_ = 0.294) or the third assessment point (*p*_C1_ = 0.648, *p*_C2_ = 0.999). However, a significant (*p*_C1_ = 0.041) improvement in the environment domain was observed in group C_1_ from the first to the third assessment point. In all other groups, there was no significant progress in the environment domain from the first to the second (*p*_A_ = 0.119, *p*_B_ = 0.658, *p*_C1_ = 0.706, *p*_C2_ = 0.710) or to the third assessment point (*p*_C2_ = 0.635).

## 4 Discussion

### 4.1 Study population and disease-specific data

Due to the increasing number of patients with PACS or PACVS and the associated long-term effects, such as cognitive deficits, there is a need for research regarding potential treatment schemes. However, to evaluate possible treatment options, it is equally essential to gain an understanding of the cognitive deficits in affected patients. This study investigated a total of *n* = 29 patients with Post-acute COVID-19 Syndrome (PACS) or Post-acute COVID-19 Vaccination Syndrome (PACVS). The primary objective of the study was to gain a better understanding of the cognitive deficits in these patients and to evaluate options for cognitive training. Therefore, this study placed additional emphasis on the deficits in cognitive performance, particularly in the domains attention, memory, and executive functions. Based on the Post-COVID-19 Functional Status Scale, 22 (75.9%) patients were categorized as grade II and 7 (24.1%) patients as grade III, indicating the need for rehabilitative and therapeutic treatment options (32). As potential treatment options to enhance cognitive performance, phosphatidylcholine and CTT were evaluated. In terms of demographic data (including age, gender distribution, disease severity, and neurological and psychiatric pre-existing conditions), this study was comparable to previous studies (10, 11, 23). Furthermore, the manifestations of neurological and psychiatric PACS and PACVS symptoms were comparable to those described in literature (10, 23). In this cohort, the majority of patients reported fatigue, word-finding difficulties, sleep disorders, and headaches, alongside the inclusion criteria for concentration disturbances and/or memory loss.

### 4.2 Patients with PACS or PACVS show cognitive deficits especially in the domain of attention

Many standardized cognitive performance test procedures, such as the MMSE, have so far proven unsuitable for evaluating cognitive deficits in PACS/PACVS (57). In several studies, mild abnormalities were observed in the Montreal Cognitive Assessment (58, 59). However, explanations for these findings remain incomplete due to limitations inherent to the testing methodology. Hence, this analysis conducted a more detailed examination using the COGBAT test set. Compared to the general population, the included patients showed deficits in general cognitive performance, mainly in the domain attention. Compared to literature, this study underlined the assumption that individuals with PACS or PACVS exhibit cognitive deficits, especially in the domains attention, memory, and executive functions (19–21, 27, 60). Memory and executive function deficits were observed, but did not differ significantly from the general population. In a positron emission tomography (PET) analysis, various brain regions exhibited hypometabolism in patients with PACS, including the orbital gyrus, olfactory gyrus, temporal lobe, amygdala, hippocampus, thalamus, brain stem, and cerebellum (61). This effect could potentially contribute to the neuropsychological symptoms observed in PACS patients. The cognitive deficits primarily affect older patients. This was also demonstrated by a significant negative correlation between age and cognitive performance in this study. Additionally, the duration of the illness and thus, a frequently occurring onset of depression play a crucial role in cognitive performance.

### 4.3 In terms of quality of life, patients with PACS/PACVS exhibit parallels with various psychiatric and chronic disorders, particularly in physical and psychological health

In this study, regarding satisfaction with their own health status, 86.2% of PACS/PACVS patients reported being very dissatisfied or dissatisfied. Based on Cronbach’s alpha coefficients *α* > 0.7, the psychological and environmental domains exhibited good internal consistency. The physical health and social relationships domains showed acceptable internal consistency with Cronbach’s alpha coefficients of 0.66 and 0.60, respectively. In the domains of physical health and psychological health, average WHOQOL-BREF scores were *M =* 41.3 ± 22.2 and 47.5 ± 14.1. These scores fell below the cut-off point of 60 (62) and were comparable to various psychiatric disorders such as major depression, eating disorders, bipolar disorder, schizophrenia, or chronic pain patients (63–68). Our study exhibited comparable results in the area of physical health to patients with myalgic encephalomyelitis/chronic fatigue (69). The average scores for the social relationships and environment domains were 64.9 ± 19.0 and 68.4 ± 20.2, indicating that these domains were above the cut-off. There were no significant relevant changes in the WHOQOL-BREF scores across the assessment time points. Therefore, no therapy recommendation could be made in our study. Furthermore, it is unsurprising that this study revealed a strong negative correlation between depression as a pre-existing condition and responses in the domains of psychological health and social relationships. Many negative responses in the psychological health domain, such as diminished enjoyment in life (Q5), negative feelings (Q6), or negative self-esteem (Q19), overlap with symptoms of depression (51). A primary symptom of depression is the neglect of social relationships, which is reflected in the responses of the WHOQOL-BREF.

Research has extensively documented a global increase in depression and anxiety levels during the COVID-19 pandemic, affecting not only PACS and/or PACVS patients (70). Consistent with prior findings, PACS/PACVS patients often exhibit cognitive deficits alongside symptoms of depression and/or anxiety (23, 71). Moreover, the duration of illness appears to be associated with the severity of these mental health issues. Furthermore, another study suggests that many of these newly reported symptoms in PACS may have predated the viral infection itself (72).

### 4.4 Patients with PACS and PACVS demonstrate improvement in their cognitive performance over time, regardless of the evaluated therapeutic scheme

PACS and PACVS patients included in our study demonstrated significant improvement in cognitive performance and in the domains of attention, memory, and executive functions. Apart from a few exceptions, this improvement was observed in all therapy schemes. Unlike previous studies, the improvement in cognitive performance cannot be attributed to cognitive training or CCT programs (73). In our study, phosphatidylcholine showed no effect on cognitive deficits in PACS and PACVS beyond the placebo effect. Since there is no significant interaction between treatment and assessment time point, it can be assumed that the improvement occurred due to other factors. The most plausible explanation for this improvement could be a spontaneous and continuous remission of cognitive deficits in PACS and PACVS, which has also been postulated as a possible course in the literature (27). This effect was also observed in other neurological PACS symptoms such as olfactory loss or fatigue (59, 74). Further, an 8-week follow-up may be insufficient to comprehensively assess the long-term effects of phosphatidylcholine and CCT. Both the neuroprotective effects of phosphatidylcholine and the enduring impact of CCT may require a longer period to fully manifest. Extended follow-up is crucial to accurately determine the stability and durability of any observed cognitive improvements, as well as to monitor for any delayed effects or adverse outcomes. In the literature, there is limited evidence regarding the temporal course of cognitive impairments. Thus far, there is a divided opinion regarding the improvement of cognitive deficits in PACS patients (75, 76). Consistent with this study, one study demonstrated improvement in the domain of complex attention (77). Moreover, the duration of PACS or PACVS in months did not correlate with improvement in COGBAT test. However, this might be due to the small sample size. Around half of the included patients reported a subjective improvement in cognitive performance. Hereby, it should be noted that the patients were aware of which treatment measures they received. It is therefore conceivable that the knowledge of receiving one or even two treatment measures may have led to a subjective improvement. Moreover, subjective improvement in cognitive performance of PACS patients does not necessarily correlate with improvement in neuropsychological testing (23).

### 4.5 Limits

The evaluation of the therapy schemes had several limitations. First of all, patients with status post critical course of SARS-CoV-2 infection or Post-COVID-19 Functional Status Scale grade IV were underrepresented in this study. The analysis did not differentiate between PACS and PACVS and the vaccination status of PACS patients was not included in the analysis due to the low number of cases. Furthermore, this analysis did not differentiate between the virus variants of the causative SARS-CoV-2 infection. It is worth noting that many laboratories in Austria did not automatically sequence the variant during PCR testing, which explains the high proportion of our patients with an unclear virus variant. Additionally, it is uncertain from the literature whether the virus variant has an impact on the severity of PACS (78–80). It is worth mentioning that the phosphatidylcholine used contains 16.4% alcohol. Given reported cases of PACS and mast cell activation syndrome, a deterioration in symptoms due to the presence of alcohol, albeit in minimal amounts, cannot be entirely ruled out (81). Furthermore, this study was not conducted as a double-blinded randomized study, resulting in a possible selection bias. Patients were always aware of which group or therapy scheme they belonged to. Therefore, within the constraints of clinical possibilities, the study design was not optimal for demonstrating actual efficacy of the treatment schemes. Moreover, it should be noted that the low sample size may introduce statistical limitations. A small sample size can lead to several issues that compromise the reliability and validity of the results. The risk of sampling bias increases, as the sample may not be representative of the global population, potentially limiting the generalizability of the findings. Additionally, reduced statistical power heightens the risk of failing to detect true effects or, conversely, falsely rejecting the null hypothesis. Small sample sizes may further complicate the identification of differences between subgroups, such as in Group C, and can result in unstable outcomes that are influenced by individual data points. Regardless of the statistical limitations, it should be noted that the observed results may have been influenced by a potential learning effect. However, in order to mitigate this risk, the COGBAT test, which has two versions to minimize the learning effects, was used as the testing procedure.

### 4.6 Conclusion

This study investigated the consequences of Post-acute COVID-19 Syndrome (PACS) and Post-acute COVID-19 Vaccination Syndrome (PACVS) on cognitive performance. Additionally, phosphatidylcholine and a CCT program were evaluated as potential treatment options. The included PACS and PACVS patients exhibited significant deficits in cognitive performance, particularly in the domain of attention, based on COGBAT testing. The effectiveness of treatment against cognitive deficits in PACS or PACVS with phosphatidylcholine and CCT could not be demonstrated, highlighting the need for further research to optimize treatment approaches. The WHOQOL-BREF primarily demonstrated deficits in the domains of physical health and psychological health, but was not impacted by the evaluated treatment schemes. However, this study provided evidence suggesting that spontaneous remission of cognitive deficits in PACS and PACVS may be possible.

## Data Availability

The raw data supporting the conclusions of this article will be made available by the authors, without undue reservation.

## References

[1] World Health Organization. WHO coronavirus (COVID-19). (2023). Available at: https://data.who.int/dashboards/covid19/cases (Accessed on 31st March 2024).

[2] National Institute for Health and Care Excellence. (2022) COVID-19 rapid guideline: managing the longterm effects of COVID-19. National Institute for Health and Care Excellence (NICE). 34181371

[3] Scholkmann F May CA. COVID-19, post-acute COVID-19 syndrome (PACS, "long COVID") and post-COVID-19 vaccination syndrome (PCVS, "post-COVIDvac-syndrome"): similarities and differences. Pathol Res Pract. (2023) 246:154497. doi: 10.1016/j.prp.2023.154497 37192595 PMC10154064

[4] Soriano JB Murthy S Marshall JC Relan P Diaz JV WHO Clinical Case Definition Working Group on Post-COVID-19 Condition. A clinical case definition of post COVID-19 condition by a Delphi consensus. Lancet Infect Dis. (2021, 2022) 22:e107:e102. doi: 10.1016/S1473-3099(21)00703-9 PMC8691845 34951953

[5] Caron P. Autoimmune and inflammatory thyroid diseases following vaccination with SARS-CoV-2 vaccines: from etiopathogenesis to clinical management. Endocrine. (2022) 78:406–17. doi: 10.1007/s12020-022-03118-4 35763241 PMC9243876

[6] Finsterer J. Neurological side effects of SARS-CoV-2 vaccinations. Acta Neurol Scand. (2022) 145:5–9. doi: 10.1111/ane.13550, PMID: 34750810 PMC8653194

[7] Rodríguez Y Rojas M Beltrán S Polo F Camacho-Domínguez L Morales SD. Autoimmune and autoinflammatory conditions after COVID-19 vaccination. New case reports and updated literature review. J Autoimmun. (2022) 132:102898. doi: 10.1016/j.jaut.2022.102898, PMID: 36041291 PMC9399140

[8] Shiravi AA Ardekani A Sheikhbahaei E Heshmat-Ghahdarijani K. Cardiovascular complications of SARS-CoV-2 vaccines: an overview. Cardiol. Ther. (2022) 11:13–21. doi: 10.1007/s40119-021-00248-0, PMID: 34845662 PMC8629102

[9] Sriwastava S Sharma K Khalid SH Bhansali S Shrestha AK Elkhooly M. COVID-19 vaccination and neurological manifestations: a review of case reports and case series. Brain Sci. (2022) 12:407. doi: 10.3390/brainsci12030407, PMID: 35326363 PMC8946610

[10] Boesl F Audebert H Endres M Prüss H Franke C. A neurological outpatient Clinic for Patients with Post-COVID-19 syndrome - a report on the clinical presentations of the first 100 patients. Front Neurol. (2021) 12:738405. doi: 10.3389/fneur.2021.738405 34603189 PMC8481602

[11] Fleischer M Szepanowski F Tovar M Herchert K Dinse H Schweda A. Post-COVID-19 syndrome is rarely associated with damage of the nervous system: findings from a prospective observational cohort study in 171 patients. Neurol Ther. (2022) 11:1637–57. doi: 10.1007/s40120-022-00395-z, PMID: 36028604 PMC9417089

[12] Moura AEF Oliveira DN Torres DM Tavares-Júnior JWL Nóbrega PR Braga-Neto P. Central hypersomnia and chronic insomnia: expanding the spectrum of sleep disorders in long COVID syndrome - a prospective cohort study. BMC Neurol. (2022) 22:417. doi: 10.1186/s12883-022-02940-7 36352367 PMC9643986

[13] Nalbandian A Sehgal K Gupta A Madhavan MV McGroder C Stevens JS. Post-acute COVID-19 syndrome. Nat Med. (2021) 27:601–15. doi: 10.1038/s41591-021-01283-z 33753937 PMC8893149

[14] Nasserie T Hittle M Goodman SN. Assessment of the frequency and variety of persistent symptoms among patients with COVID-19: a systematic review. JAMA Netw Open. (2021) 4:e2111417. doi: 10.1001/jamanetworkopen.2021.11417 34037731 PMC8155823

[15] Premraj L Kannapadi NV Briggs J Seal SM Battaglini D Fanning J. Mid and long-term neurological and neuropsychiatric manifestations of post-COVID-19 syndrome: a meta-analysis. J Neurol Sci. (2022) 434:120162. doi: 10.1016/j.jns.2022.120162, PMID: 35121209 PMC8798975

[16] Salamanna F Veronesi F Martini L Landini MP Fini M. Post-COVID-19 syndrome: the persistent symptoms at the post-viral stage of the disease. A Systematic Review of the Current Data. Front Med (Lausanne). (2021) 8:653516. doi: 10.3389/fmed.2021.653516 34017846 PMC8129035

[17] Wulf Hanson S Abbafati C Aerts JG Al-Aly Z Ashbaugh C Ballouz T. A global systematic analysis of the occurrence, severity, and recovery pattern of long COVID in 2020 and 2021. medRxiv. (2022) 27:2022.05.26.22275532. doi: 10.1101/2022.05.26.22275532

[18] Funke K Bernard M Luppa M Riedel-Heller SG Luck T. Impaired activities of daily living in neurocognitive disorders: development of a complex asse:ssment for research and practice. Nervenarzt. (2022) 93:840–3. doi: 10.1007/s00115-021-01257-z 35006296 PMC9363366

[19] Bertuccelli M Ciringione L Rubega M Bisiacchi P Masiero S Del Felice A. Cognitive impairment in people with previous COVID-19 infection: a scoping review. Cortex. (2022) 154:212–30. doi: 10.1016/j.cortex.2022.06.002 35780756 PMC9187867

[20] Delgado-Alonso C Valles-Salgado M Delgado-Álvarez A Yus M Gómez-Ruiz N Jorquera M. Cognitive dysfunction associated with COVID-19: a comprehensive neuropsychological study. J Psychiatr Res. (2022) 150:40–6. doi: 10.1016/j.jpsychires.2022.03.033, PMID: 35349797 PMC8943429

[21] García-Sánchez C Calabria M Grunden N Pons C Arroyo JA Gómez-Anson B. Neuropsychological deficits in patients with cognitive complaints after COVID-19. Brain Behav. (2022) 12:e2508. doi: 10.1002/brb3.2508, PMID: 35137561 PMC8933779

[22] Hadad R Khoury J Stanger C Fisher T Schneer S Ben-Hayun R. Cognitive dysfunction following COVID-19 infection. J Neurovirol. (2022) 28:430–7. doi: 10.1007/s13365-022-01079-y, PMID: 35618983 PMC9134977

[23] Ludwig B Deckert M Krajnc N Keritam O Macher S Bsteh G. Reported neurological symptoms after severe acute respiratory syndrome coronavirus type 2 infection: a systematic diagnostic approach. Eur J Neurol. (2023) 30:2713–25. doi: 10.1111/ene.15923, PMID: 37306533

[24] Matias-Guiu JA Herrera E González-Nosti M Krishnan K Delgado-Alonso C Díez-Cirarda M. Development of criteria for cognitive dysfunction in post-COVID syndrome: the IC-CoDi-COVID approach. Psychiatry Res. (2023) 319:115006. doi: 10.1016/j.psychres.2022.115006, PMID: 36521337

[25] Serrano-Castro P Garzón-Maldonado F Casado-Naranjo I Ollero-Ortiz A Mínguez-Castellanos A Iglesias-Espinosa M. The cognitive and psychiatric subacute impairment in severe Covid-19. Sci Rep. (2022) 12:3563. doi: 10.1038/s41598-022-07559-9 35241761 PMC8894467

[26] Shanley JE Valenciano AF Timmons G Miner AE Kakarla V Rempe T. Longitudinal evaluation of neurologic-post acute sequelae SARS-CoV-2 infection symptoms. Ann Clin Transl Neurol. (2022) 9:995–1010. doi: 10.1002/acn3.51578, PMID: 35702954 PMC9268882

[27] Schilling C Meyer-Lindenberg A Schweiger JI. Cognitive disorders and sleep disturbances in long COVID. Nervenarzt. (2022) 93:779–87. doi: 10.1007/s00115-022-01297-z. 35576015 PMC9109661

[28] Zamponi HP Juarez-Aguaysol L Kukoc G Dominguez ME Pini B Padilla EG. Olfactory dysfunction and chronic cognitive impairment following SARS-CoV-2 infection in a sample of older adults from the Andes mountains of Argentina. Alzheimers Dement. (2021) 17:e057897. doi: 10.1002/alz.057897

[29] Whiteside DM Naini SM Basso MR Waldron EJ Holker E Porter J. Outcomes in post-acute sequelae of COVID-19 (PASC) at 6 months post-infection part 2: psychological functioning. Clin Neuropsychol. (2022) 36:829–47. doi: 10.1080/13854046.2022.2030411, PMID: 35098861

[30] Barker-Davies RM O'Sullivan O Senaratne KPP Baker P Cranley M Dharm-Datta S. The Stanford hall consensus statement for post-COVID-19 rehabilitation. Br J Sports Med. (2020) 54:949–59. doi: 10.1136/bjsports-2020-102596, PMID: 32475821 PMC7418628

[31] Sivan M Rayner C Delaney B. Fresh evidence of the scale and scope of long covid. BMJ. (2021) 373:n853. doi: 10.1136/bmj.n853 33795224

[32] Klok FA Boon G Barco S Endres M Geelhoed JJM Knauss S. The post-COVID-19 functional status scale: a tool to measure functional status over time after COVID-19. Eur Respir J. (2020) 56:2001494. doi: 10.1183/13993003.01494-2020, PMID: 32398306 PMC7236834

[33] Liu K Zhang W Yang Y Zhang J Li Y Chen Y. Respiratory rehabilitation in elderly patients with COVID-19: a randomized controlled study. Complement Ther Clin Pract. (2020) 39:101166. doi: 10.1016/j.ctcp.2020.101166 32379637 PMC7118596

[34] Puchner B Sahanic S Kirchmair R Pizzini A Sonnweber B Wöll E. Beneficial effects of multi-disciplinary rehabilitation in postacute COVID-19: an observational cohort study. Eur J Phys Rehabil Med. (2021) 57:189–98. doi: 10.23736/S1973-9087.21.06549-7, PMID: 33448756

[35] Zifko UA Yacob M Braun BJ Dietz GPH. Alleviation of post-COVID-19 cognitive deficits by treatment with EGb 761®: a case series. Am J Case Rep. (2022) 23:e937094. doi: 10.12659/AJCR.937094 36156538 PMC9523733

[36] Dysken MW Fovall P Harris CM Davis JM Noronha A. Lecithin administration in Alzheimer dementia. Neurology. (1982) 32:1203–4. doi: 10.1212/wnl.32.10.1203 6889709

[37] Latifi S Tamayol A Habibey R Sabzevari R Kahn C Geny D. Natural lecithin promotes neural network complexity and activity. Sci Rep. (2016) 6:25777. doi: 10.1038/srep25777, PMID: 27228907 PMC4882550

[38] Amenta F Parnetti L Gallai V Wallin A. Treatment of cognitive dysfunction associated with Alzheimer's disease with cholinergic precursors. Ineffective treatments or inappropriate approaches? Mech Ageing Dev. (2001) 122:2025–40. doi: 10.1016/s0047-6374(01)00310-4 11589920

[39] De Jesus Moreno Moreno M. Cognitive improvement in mild to moderate Alzheimer's dementia after treatment with the acetylcholine precursor choline alfoscerate: a multicenter, double-blind, randomized, placebo-controlled trial. Clin Ther. (2003) 25:178–93. doi: 10.1016/S0149-2918(03)90023-3, PMID: 12637119

[40] Di Perri R Coppola G Ambrosio LA Grasso A Puca FM Rizzo M. A multicentre trial to evaluate the efficacy and tolerability of alpha-glycerylphosphorylcholine versus cytosine diphosphocholine in patients with vascular dementia. J Int Med Res. (1991) 19:330–41. doi: 10.1177/030006059101900406 1916007

[41] Moré MI Freitas U Rutenberg D. Positive effects of soy lecithin-derived phosphatidylserine plus phosphatidic acid on memory, cognition, daily functioning, and mood in elderly patients with Alzheimer's disease and dementia. Adv Ther. (2014) 31:1247–62. doi: 10.1007/s12325-014-0165-1 25414047 PMC4271139

[42] Parnetti L Amenta F Gallai V. Choline alphoscerate in cognitive decline and in acute cerebrovascular disease: an analysis of published clinical data. Mech Ageing Dev. (2001) 122:2041–55. doi: 10.1016/s0047-6374(01)00312-8 11589921

[43] Shi F Zhou J Meng D. Curative effect of soybean lecithin on cerebral infarction. Zhonghua Yi Xue Za Zhi. (2001) 81:1301–3. 16200721

[44] Patrick RP. Role of phosphatidylcholine-DHA in preventing APOE4-associated Alzheimer's disease. FASEB J. (2019) 33:1554–64. doi: 10.1096/fj.201801412R, PMID: 30289748 PMC6338661

[45] Stark BC Warburton EA. Improved language in chronic aphasia after self-delivered iPad speech therapy. Neuropsychol Rehabil. (2018) 28:818–31. doi: 10.1080/09602011.2016.1146150 26926872

[46] De Luca R Leonardi S Spadaro L Russo M Aragona B Torrisi M. Improving cognitive function in patients with stroke: can computerized training be the future? J Stroke Cerebrovasc Dis. (2018) 27:1055–60. doi: 10.1016/j.jstrokecerebrovasdis.2017.11.008, PMID: 29221967

[47] Fridriksson J Baker JM Whiteside J Eoute D Jr Moser D Vesselinov R. Treating visual speech perception to improve speech production in nonfluent aphasia. Stroke. (2009) 40:853–8. doi: 10.1161/STROKEAHA.108.532499 19164782 PMC2679690

[48] Katz RC Wertz RT. The efficacy of computer-provided reading treatment for chronic aphasic adults. J Speech Lang Hear Res. (1997) 40:493–507. doi: 10.1044/jslhr.4003.493 9210109

[49] Palmer R Enderby P Cooper C Latimer N Julious S Paterson G. Computer therapy compared with usual care for people with long-standing aphasia poststroke: a pilot randomized controlled trial. Stroke. (2012) 43:1904–11. doi: 10.1161/STROKEAHA.112.650671, PMID: 22733794

[50] Skevington SM Lotfy M O'Connell KA. The World Health Organization's WHOQOL-BREF quality of life assessment: psychometric properties and results of the international field trial. A report from the WHOQOL group. Qual Life Res. (2004) 13:299–310. doi: 10.1023/B:QURE.0000018486.91360.00, PMID: 15085902

[51] The WHOQOL Group. Development of the World Health Organization WHOQOL-BREF quality of life assessment. Psychol Med. (1998) 28:551–8. doi: 10.1017/S0033291798006667 9626712

[52] Aschenbrenner S Kaiser S Pfüller U Roesch-Ely D Weisbrod M. Wiener Testsystem: Testset Kognitive Basistestung (CogBat). Mödling: Schuhfried (2012).

[53] Petersen SE van Mier H Fiez JA Raichle ME. The effects of practice on the functional anatomy of task performance. Proc Natl Acad Sci USA. (1998) 95:853–60. doi: 10.1093/cercor/4.1.8 9448251 PMC33808

[54] Kim YH Yoo WK Ko MH Park CH Kim ST Na DL. Plasticity of the attentional network after brain injury and cognitive rehabilitation. Neurorehabil Neural Repair. (2009) 23:468–77. doi: 10.1177/1545968308328728 19118131

[55] Sturm W Longoni F Weis S Specht K Herzog H Vohn R. Functional reorganisation in patients with right hemisphere stroke after training of alertness: a longitudinal PET and fMRI study in eight cases. Neuropsychologia. (2004) 42:434–50. doi: 10.1016/j.neuropsychologia.2003.09.001 14728918

[56] Platz T Berlit P Dohle C Fickenscher H Guha M Köllner V. S2k-guideline SARS-CoV-2, COVID-19 and (early) rehabilitation - a consensus-based guideline for Germany. GMS Hyg Infect Control. (2023) 18:Doc12. doi: 10.3205/dgkh000438 37261059 PMC10227492

[57] Lauria A Carfì A Benvenuto F Bramato G Ciciarello F Rocchi S. Neuropsychological measures of post-COVID-19 cognitive status. Front Psychol. (2023) 14:1136667. doi: 10.3389/fpsyg.2023.1136667, PMID: 37492442 PMC10363721

[58] Crivelli L Palmer K Calandri I Guekht A Beghi E Carroll W. Changes in cognitive functioning after COVID-19: a systematic review and meta-analysis. Alzheimers Dement. (2022) 18:1047–66. doi: 10.1002/alz.12644, PMID: 35297561 PMC9073922

[59] Hartung TJ Neumann C Bahmer T Chaplinskaya-Sobol I Endres M Geritz J. Fatigue and cognitive impairment after COVID-19: a prospective multicentre study. EClinicalMedicine. (2022) 53:101651. doi: 10.1016/j.eclinm.2022.101651, PMID: 36133318 PMC9482331

[60] Tavares-Júnior JWL de Souza ACC Borges JWP Oliveira DN Siqueira-Neto JI Sobreira-Neto MA. COVID-19 associated cognitive impairment: a systematic review. Cortex. (2022) 152:77–97. doi: 10.1016/j.cortex.2022.04.006 35537236 PMC9014565

[61] Guedj E Campion JY Dudouet P Kaphan E Bregeon F Tissot-Dupont H. 18F-FDG brain PET hypometabolism in patients with long COVID. Eur J Nucl Med Mol Imaging. (2021) 48:2823–33. doi: 10.1007/s00259-021-05215-4, PMID: 33501506 PMC7837643

[62] Silva PA Soares SM Santos JF Silva LB. Cut-off point for WHOQOL-bref as a measure of quality of life of older adults. Rev Saude Publica. (2014) 48:390–7. doi: 10.1590/S0034-8910.2014048004912 25119934 PMC4203073

[63] Baiano M Salvo P Righetti P Cereser L Baldissera E Camponogara I. Exploring health-related quality of life in eating disorders by a cross-sectional study and a comprehensive review. BMC Psychiatry. (2014) 14:165. doi: 10.1186/1471-244X-14-165 24898768 PMC4058000

[64] Berlim MT Pavanello DP Caldieraro MA Fleck MP. Reliability and validity of the WHOQOL BREF in a sample of Brazilian outpatients with major depression. Qual Life Res. (2005) 14:561–4. doi: 10.1007/s11136-004-4694-y 15892446

[65] Gamage N Senanayake S Kumbukage M Mendis J Jayasekara A. The prevalence of anxiety and its association with the quality of life and illness severity among bipolar affective disorder patients in a developing country. Asian J Psychiatr. (2020) 52:102044. doi: 10.1016/j.ajp.2020.102044 32344280

[66] Khafif TC Belizario GO Silva M Gomes BC Lafer B. Quality of life and clinical outcomes in bipolar disorder: an 8-year longitudinal study. J Affect Disord. (2021) 278:239–43. doi: 10.1016/j.jad.2020.09.061 32971316

[67] Naumann VJ Byrne GJ. WHOQOL-BREF as a measure of quality of life in older patients with depression. Int Psychogeriatr. (2004) 16:159–73. doi: 10.1017/s1041610204000109 15318762

[68] Skevington SM McCrate FM. Expecting a good quality of life in health: assessing people with diverse diseases and conditions using the WHOQOL-BREF. Health Expect. (2012) 15:49–62. doi: 10.1111/j.1369-7625.2010.00650.x 21281412 PMC5060606

[69] Brittain E Muirhead N Finlay AY Vyas J. Myalgic encephalomyelitis/chronic fatigue syndrome (ME/CFS): major impact on lives of both patients and family members. Medicina. (2021) 57:43. doi: 10.3390/medicina57010043 33430175 PMC7825605

[70] COVID-19 Mental Disorders Collaborators. Global prevalence and burden of depressive and anxiety disorders in 204 countries and territories in 2020 due to the COVID-19 pandemic. Lancet. (2021) 398:1700–12. doi: 10.1016/S0140-6736(21)02143-7, PMID: 34634250 PMC8500697

[71] Gulpers BJA Verhey FRJ Eussen S Schram MT de Galan BE van Boxtel MPJ. Anxiety and cognitive functioning in the Maastricht study: a cross-sectional population study. J Affect Disord. (2022) 319:570–9. doi: 10.1016/j.jad.2022.09.072, PMID: 36162695

[72] Ballering AV van Zon SKR Olde Hartman TC Rosmalen JGM. Persistence of somatic symptoms after COVID-19 in the Netherlands: an observational cohort study. Lancet. (2022) 400:452–61. doi: 10.1016/S0140-6736(22)01214-4 35934007 PMC9352274

[73] Ge S Zhu Z Wu B McConnell ES. Technology-based cognitive training and rehabilitation interventions for individuals with mild cognitive impairment: a systematic review. BMC Geriatr. (2018) 18:213. doi: 10.1186/s12877-018-0893-1 30219036 PMC6139138

[74] Boscolo-Rizzo P Borsetto D Fabbris C Spinato G Frezza D Menegaldo A. Evolution of altered sense of smell or taste in patients with mildly symptomatic COVID-19. JAMA Otolaryngol Head Neck Surg. (2020) 146:729–32. doi: 10.1001/jamaoto.2020.1379, PMID: 32614442 PMC7333173

[75] Andrei Appelt P Taciana Sisconetto A Baldo Sucupira KSM Neto EM Chagas TJ Bazan R. Changes in electrical brain activity and cognitive functions following mild to moderate COVID-19: a one-year prospective study after acute infection. Clin EEG Neurosci. (2022) 53:543–57. doi: 10.1177/15500594221103834 35635280 PMC9157278

[76] Miskowiak KW Fugledalen L Jespersen AE Sattler SM Podlekareva D Rungby J. Trajectory of cognitive impairments over 1 year after COVID-19 hospitalisation: pattern, severity, and functional implications. Eur Neuropsychopharmacol. (2022) 59:82–92. doi: 10.1016/j.euroneuro.2022.04.004 35561540 PMC9008126

[77] Poletti S Palladini M Mazza MG De Lorenzo R Furlan R Ciceri F. Long-term consequences of COVID-19 on cognitive functioning up to 6 months after discharge: role of depression and impact on quality of life. Eur Arch Psychiatry Clin Neurosci. (2022) 272:773–82. doi: 10.1007/s00406-021-01346-9 34698871 PMC8546751

[78] Canas LS Molteni E Deng J Sudre CH Murray B Kerfoot E. Profiling post-COVID-19 condition across different variants of SARS-CoV-2: a prospective longitudinal study in unvaccinated wild-type, unvaccinated alpha-variant, and vaccinated delta-variant populations. Lancet Digit Health. (2023) 5:e421–34. doi: 10.1016/S2589-7500(23)00056-0, PMID: 37202336 PMC10187990

[79] Fernández-de-Las-Peñas C Cancela-Cilleruelo I Rodríguez-Jiménez J Gómez-Mayordomo V Pellicer-Valero OJ Martín-Guerrero JD. Associated-onset symptoms and post-COVID-19 symptoms in hospitalized COVID-19 survivors infected with Wuhan, alpha or Delta SARS-CoV-2 variant. Pathogens. (2022) 11:725. doi: 10.3390/pathogens11070725, PMID: 35889971 PMC9320021

[80] Magnusson K Kristoffersen DT Dell'Isola A Kiadaliri A Turkiewicz A Runhaar J. Post-covid medical complaints following infection with SARS-CoV-2 omicron vs Delta variants. Nat Commun. (2022) 13:7363. doi: 10.1038/s41467-022-35240-2, PMID: 36450749 PMC9709355

[81] Afrin LB Weinstock LB Molderings GJ. Covid-19 hyperinflammation and post-Covid-19 illness may be rooted in mast cell activation syndrome. Int J Infect Dis. (2020) 100:327–32. doi: 10.1016/j.ijid.2020.09.016, PMID: 32920235 PMC7529115

